# Challenges and Opportunities for Centers on Aging: Part 1 - Leadership, Diversity, and Advocacy

**DOI:** 10.1093/geroni/igaf122.609

**Published:** 2025-12-31

**Authors:** Pamela Saunders

## Abstract

The current national landscape poses significant challenges for the future of socio-biomedical research in the US. Politicians seek to restructure the funding priorities for essential programs that support older adults, including Medicare and organizations like NIH and the National Science Foundation. As a result, leaders at aging centers are evaluating their programs, services, structures, and sources of support. This symposium is part one of a two-part series titled “Challenges and Opportunities for Centers on Aging” and addresses issues of diversity, advocacy, mentorship, and development. The first paper will discuss recommendations and lessons learned from the startup of the new Center for Healthy Aging at Georgetown University. The second paper will explore the pivotal role of the CU Anschutz Multidisciplinary Center on Aging in leading advocacy efforts for older adults and the stakeholders who support their care. The third paper will introduce a new initiative called the Leadership Excellence and Advancement of Directors (LEAD) Program, which aims to provide mentorship, professional growth, and peer support for center directors. The fourth paper will investigate academic, professional, and policy frames of equity and social justice for the Center for the Advanced Study of Aging Services, housed in UC Berkeley’s School of Social Welfare. The fifth paper will explore strategies for overcoming financial and institutional constraints through the Marymount Center for Optimal Aging’s interdisciplinary strategic model. This model is designed to impact older adults’ well-being while providing students with hands-on training. The symposium offers recommendations to tackle challenges and seize opportunities facing aging centers.

